# Supplementary material for: The matrix vesicle cargo miR-125b accumulates in the bone matrix, inhibiting bone resorption in mice
Source: Commun Biol. 2020 Jan 16;3:30. doi: 10.1038/s42003-020-0754-2 (PMC6965124; doi:10.1038/s42003-020-0754-2)
Supplement: Supplementary file 1 — Supplementary Information [file 42003_2020_754_MOESM1_ESM.pdf]

Supplementary Figures

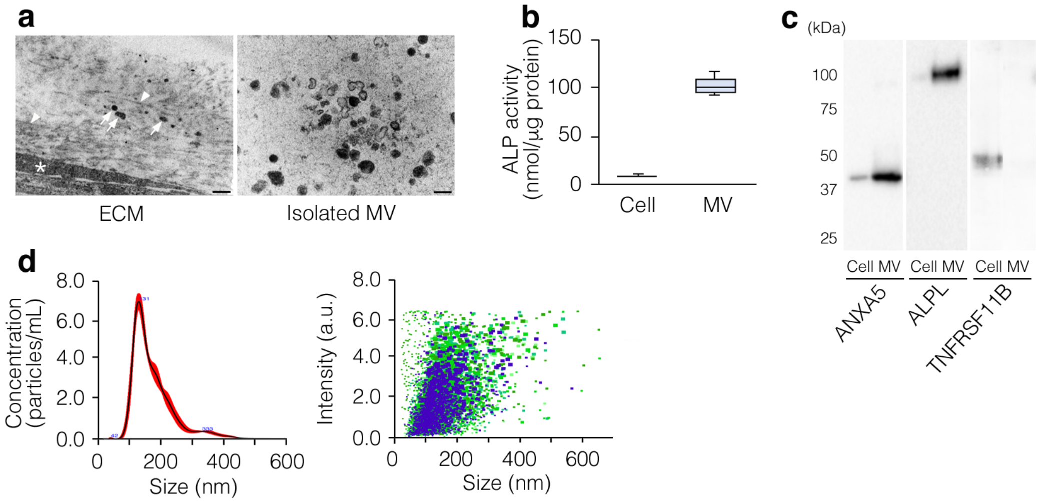

Supplementary Figure 1. Matrix vesicles (MVs) isolated from the ECM of MC3T3-E1 cell cultures. Cells were cultured until osteoid-like nodules were formed. The cell and MV fractions were isolated by collagenase digestion in combination with ultracentrifugation. (**a**) Transmission electron microscope images showing MVs in the ECM with cells and isolated MVs (representative images of three independent experiments). *Arrows* and *arrowheads* indicate MVs and collagen fibers, respectively. *Asterisk* shows cells. Scale bars, 500 nm (left) and 200 nm (right). (**b**) Alkaline phosphatase (ALP) activity of cells and MVs (n=3). (**c)** Expression of ANXA5, ALPL and TNFRSF-11B (representative blotting of three independent experiments) is shown. MVs, 2 μg protein; cells, 10 μg protein. (**d**) Size distribution (concentrations and intensities) of isolated MVs (representative data sets of three independent experiments). MVs (5 μL stock solution) were diluted with PBS and analyzed by NanoSight NS300.

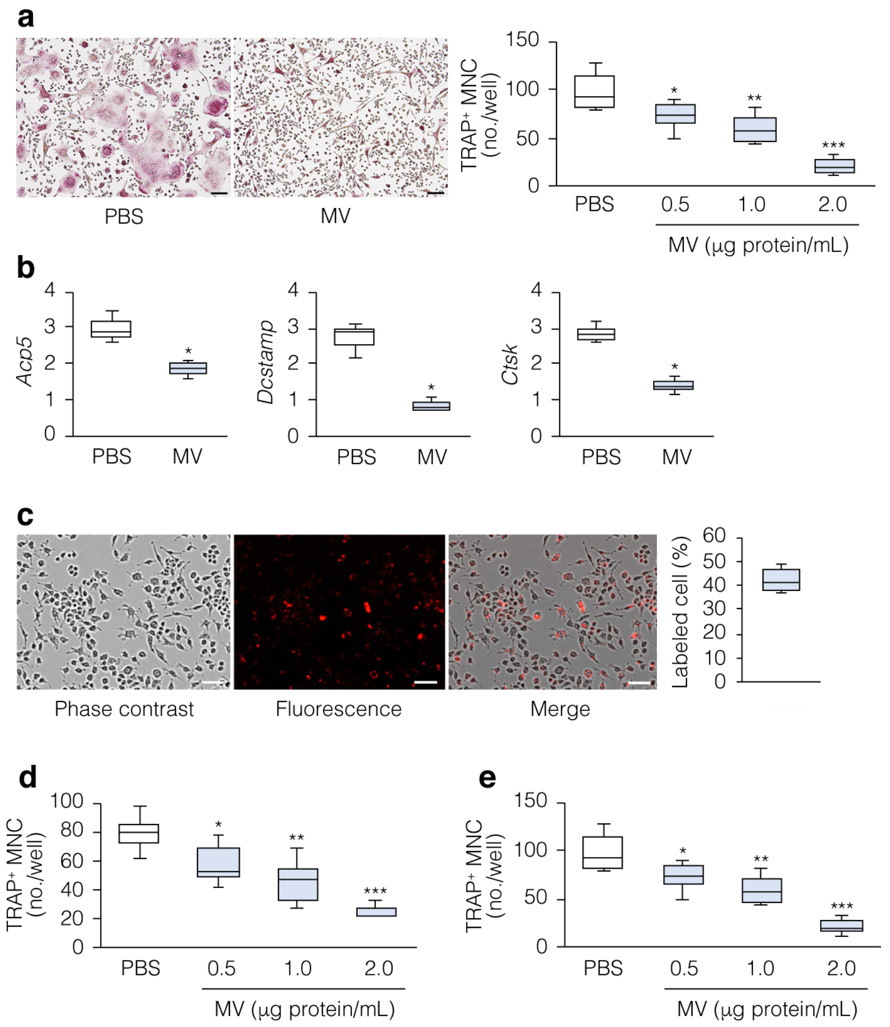

Supplementary Figure 2. MVs inhibit TRAP$^+$ multinucleated cell (MNC) formation in RAW-D cell cultures. Cells were stimulated with RANKL. (**a**) Representative images of TRAP$^+$ MNC formation and the number of TRAP$^+$ MNCs/well with MVs at indicated concentrations or PBS (*n*=6). (**b**) Expression of osteoclast marker genes, *Acp5*, *Dcstamp*, and *Ctsk* (*n*=3). *Rpl32* was used as internal control. Cells were treated with MVs (1μg protein/mL) or PBS. (**c**) Representative images of incorporation of MVs labeled with lipophilic tracer DiI (2 μg protein/mL) into cells and the percentage of labeled cells (*n*=6). (**d**,**e**) The number of TRAP$^+$MNCs/well with or without rat (**d**) and human (**e**) MVs at indicated concentrations (*n*=6). Scale bars, 100 μm. **P*<0.05, ***P*<0.01 and ****P*<0.001 vs. PBS (-) by Tukey's multiple comparison (**a**,**d**,**e**) and by Student's *t*-test (**b**).

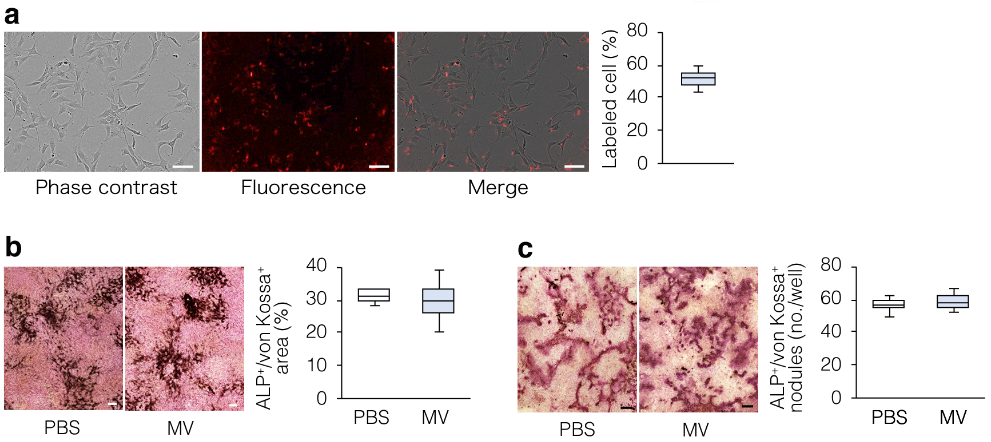

Supplementary Figure 3. MVs have no effect on osteoblast development in vitro. MC3T3-E1 cells (**a**,**b**) and rat primary osteoblasts (**c**) were treated with MVs (2 μg protein/mL) or PBS under osteogenic conditions. (**a**) Representative images of incorporation of MVs labeled with lipophilic tracer DiI into cells and the percentage of labeled cells (*n*=6). (**b**,**c**) Representative images of ALP/von Kossa staining showing osteoblast development and the percentage of ALP^{+}/von $Kossa^{+}$ areas (**b**) and the number (**c**) of ALP^{+}/von $Kossa^{+}$ nodules (*n*=4). Scale bars, 100 μm (**a**) and 500 μm (**b**,**c**).

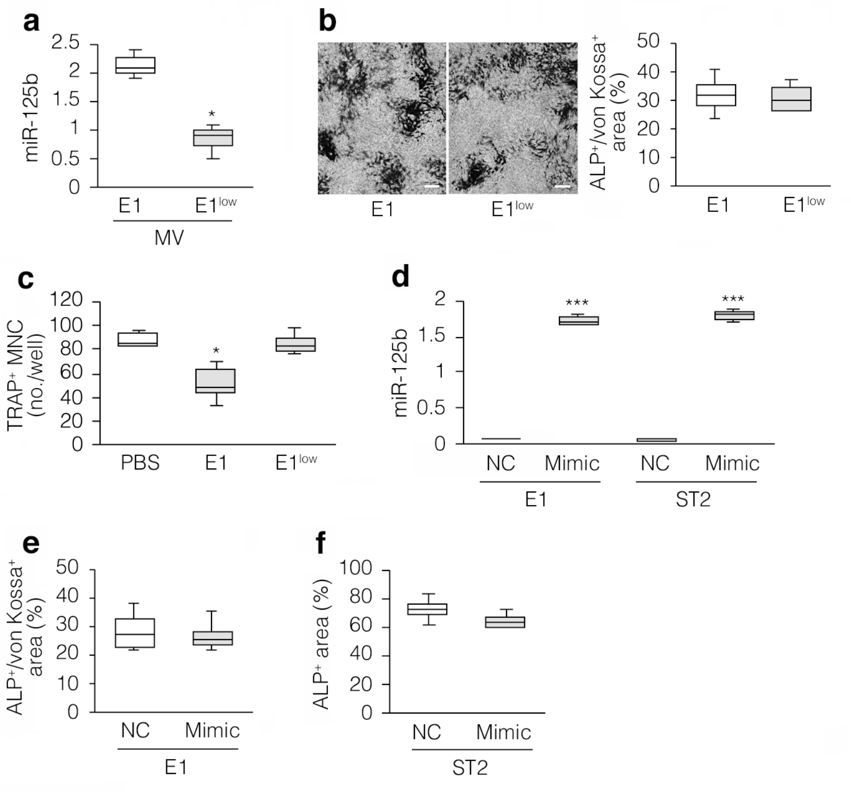

Supplementary Figure 4. miR-125b levels are not implicated in osteoblast development in vitro. MC3T3-E1 (E1) cells and E1^{low} cells (the MC3T3-E1 subclone) were cultured under osteogenic conditions. (**a**) miR-125b levels in MVs; U6 was used as internal control (*n*=3). (**b**) Representative images of ALP/von Kossa staining and the percentage of ALP^{+}/vonKossa^{+} areas (*n*=4). Scaler bars, 500 µm. (**C**) The number of TRAP^{+} multinucleated cells (MNCs)/well in RAW-D cell cultures treated with E1^{low} MVs, E1 MVs (1 µg protein/mL each) or PBS (*n*=6). RAW-D cells were stimulated with RANKL. (**d**) miR-125b levels in E1 cells and ST2 cells transfected with miR-125b mimic (Mimic) or negative control miRNA (NC); U6 was used as internal control (*n*=3). (**e**,**f**) The percentage of ALP/von Kossa^{+} areas in E1 cell cultures and ALP^{+} areas in ST2 cell cultures, when transfected with Mimic or NC (*n*=4). *P<0.05 and ***P<0.001 vs. matched control by Student's *t*-test (**b**,**d**) and by Tukey's multiple comparison (**c**).

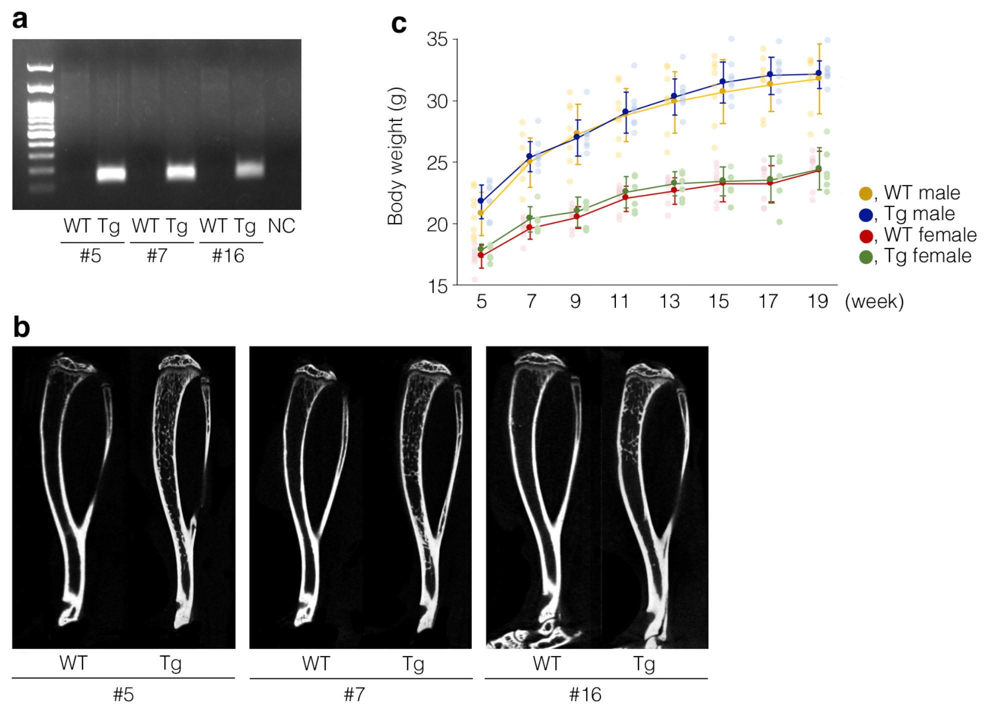

Supplementary Figure 5. Three transgenic (Tg) mouse lines expressing miR-125b in osteoblasts have increased trabecular bones. (**a**) Representative agarose gel electrophoresis of a PCR product of transgene in 9-week-old male Tg lines #5 and #7 and 12-week-old male Tg line #16, and corresponding wild type (WT) littermates (representative images of 5 mice in each genotype). NC, Negative control for PCR. A 100 bp DNA size marker is shown. (**b**) Representative 2D images of micro computed tomography (μCT) of tibiae of three mouse lines (representative images of 5 mice in each genotype). (**c**) Growth curves of #5 male and female Tg and WT mice. Results are presented as mean ± SD. *n*=6.

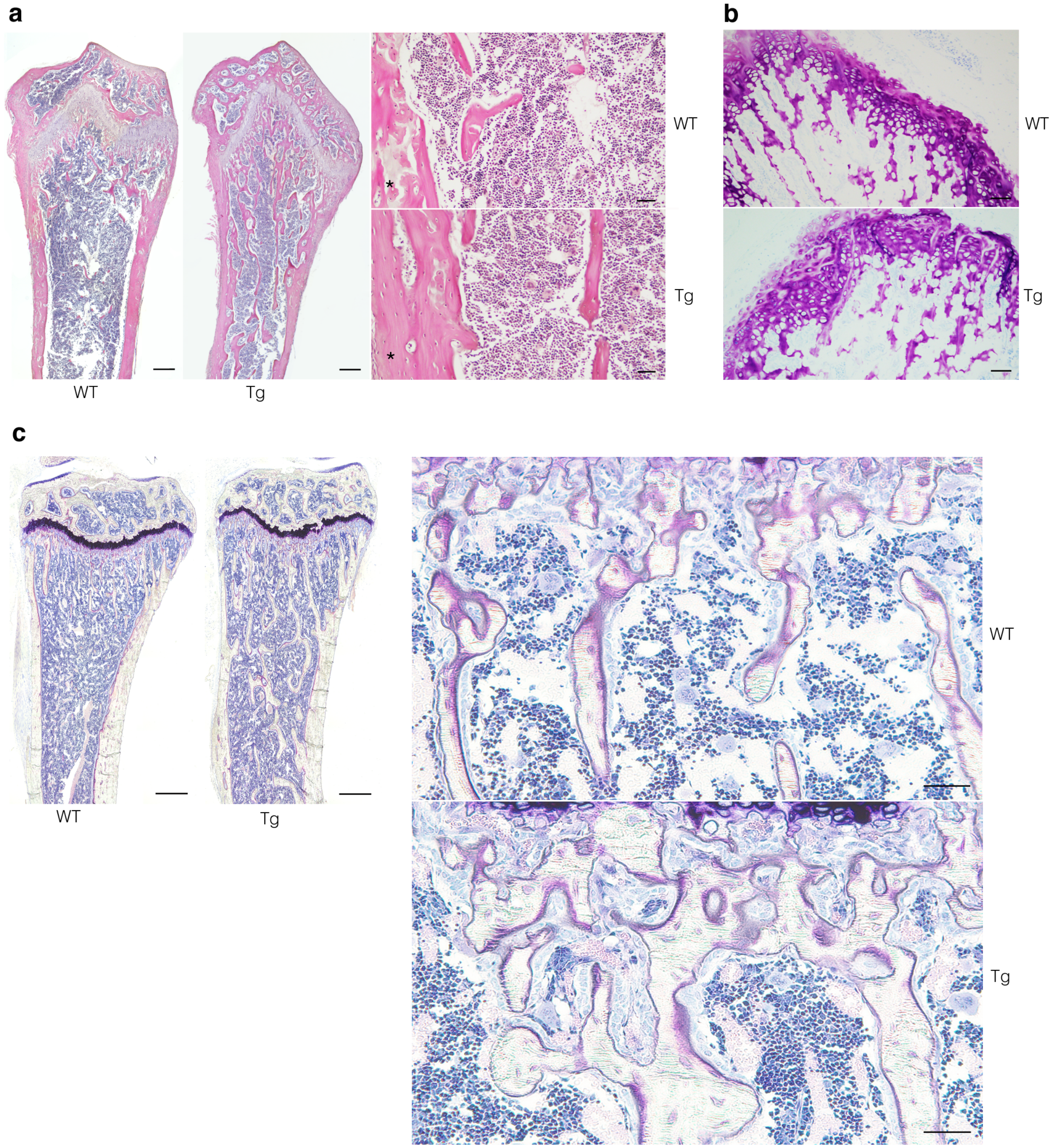

Supplementary Figure 6. **Histological analysis of the Tg and WT femurs and tibiae.** Demineralized paraffin sections (**a**,**b**; 5 μm thickness) of the distal femurs and plastic sections (**c**; 4 μm thickness) of proximal tibiae were prepared from 9-week-old and 10-week-old female mice, respectively. (**a**) H&E staining; low magnification images (left panels; scale bars, 250 μm) and bone marrow cells with cortical bone (*) at high magnification (right panels; scale bars, 50 μm). (**b**) Growth plates stained with toluidine blue. Scale bars, 50 μm. (**c**) Villanueva staining; low magnification images (left panels; scale bars, 250 μm) and primary spongiosa at high

magnification (right panels; scale bars, 50 μm). All images are representative of 5 mice in each phenotype.

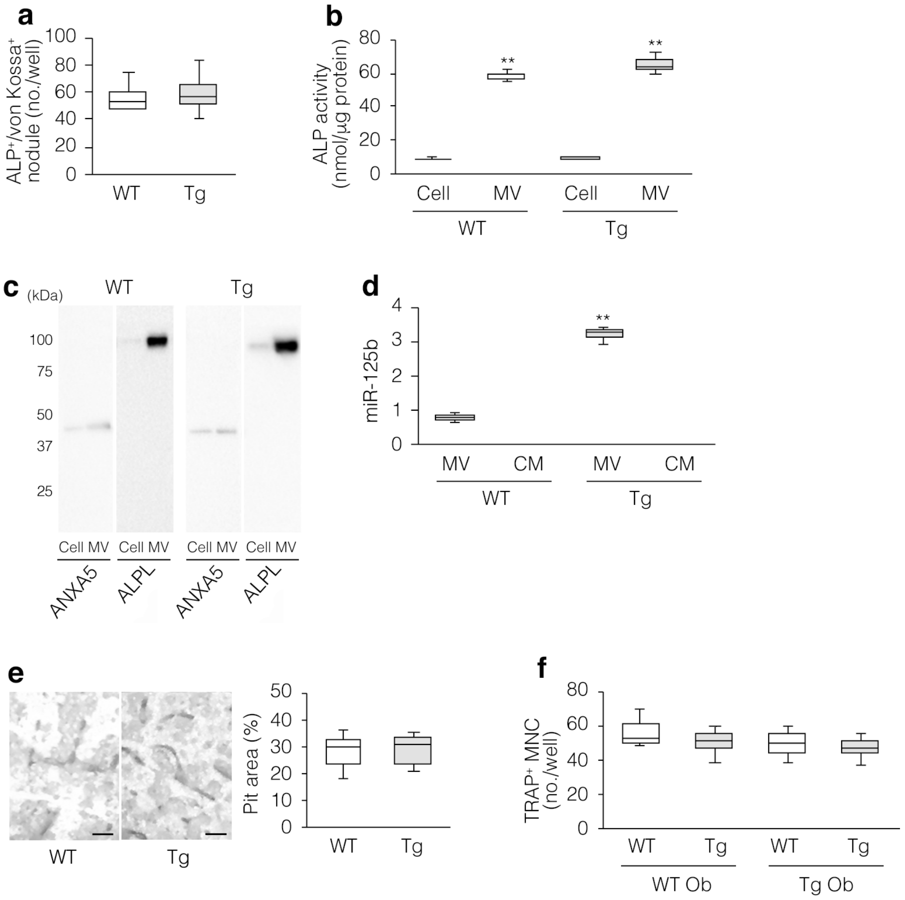

Supplementary Figure 7. Characteristics of Tg and WT calvaria osteoblasts and BMMs. Calvaria cells isolated from newborn mice were cultured under osteogenic conditions. The cell and MV fractions were isolated and conditioned media (CM) were collected, when osteoid-like nodules formed. (**a**) The number of ALP/von Kossa$^+$ nodules/well measured using ImageJ (*n*=4). (**b**) ALP activities in cells and MVs (*n*=3). (**c**) Expression of ALPL and ANXA5 in cells (10 µg protein) and MVs (2 µg protein) (Representative blotting of three independent experiments). (**d**) miR-125b levels in MVs and CM; U6 (MVs) and miR16 (CM) were used as internal controls (*n*=3). (**e**) Representative images of resorption pits (scale bars, 50 µm) and the percent of Pit areas. (**f**) The number of TRAP$^+$ multinucleated cells (MNCs) of either Tg or WT BMMs cocultured with Tg vs. WT osteoblasts (Ob). BMMs obtained from 9-week-old male mice (*n*=6). Obs forming osteoid-like nodules were derived from newborn mouse calvariae (*n*=6). 1,25-dihydrixyvitamin D_3 and prostaglandin E_2 were used to induce osteoclastogenesis. $^{**}P<0.01$ vs. matched control by Student's *t*-test (**b**,**d**).

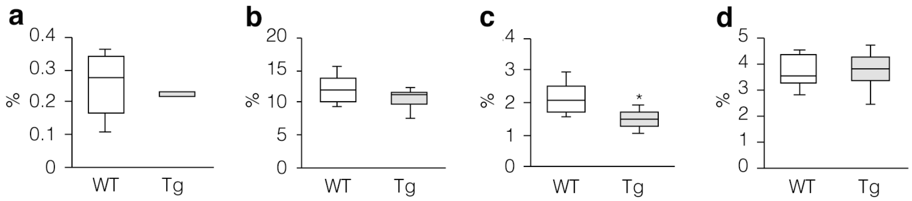

Supplementary Figure 8. **FACS analysis of bone marrow cells in 8-week-old Tg and WT male mice.** The percentages of hematopoietic stem cells (**a**), B cells (**b**), T cells (**c**) and macrophages (**d**) are shown (*n*=8). *P<0.05 by Student's *t*-test.

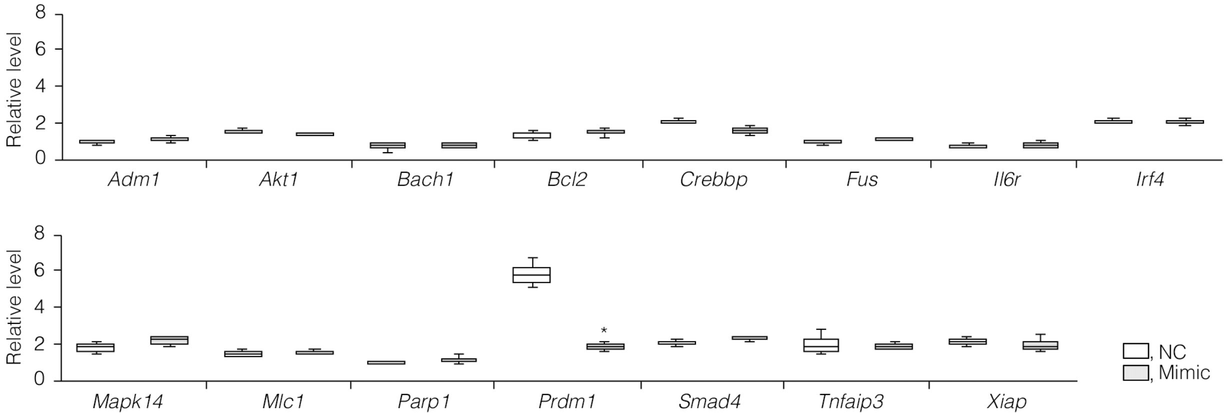

Supplementary Figure 9. *Prdm1* is downregulated in RAW-D cells transfected with miR-125b mimic. Cells were transfected with miR-125b mimic (Mimic) or negative control miRNA (NC) in the presence of RANKL for 48 h; *Rpl32* was used as internal control (n=3). *P<0.05 by Student's t-test.

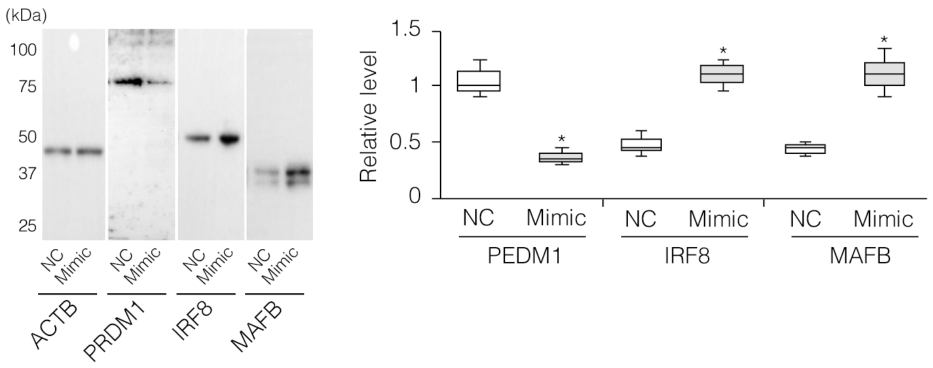

Supplementary Figure 10. **Western blot analysis of PRDM1, IRF8, and MAFB in RAW-D cells transfected with miR-125b mimic.** Cell transfected with miR-125b mimic (Mimic) or negative control miRNA (NC) in the presence of RANKL for 48 h were lysed for Western blotting analysis. Representative blotting of three independent experiments. Relative expression levels are presented (*n*=3). The intensities of NC in PRDM1 and of Mimic in MAFB and IRF8 were set at 1.0. ACTB was used as internal control. Aliquots of cell lysate (10 µg protein) were used for analysis. **P*<0.05 by Student's *t*-test.

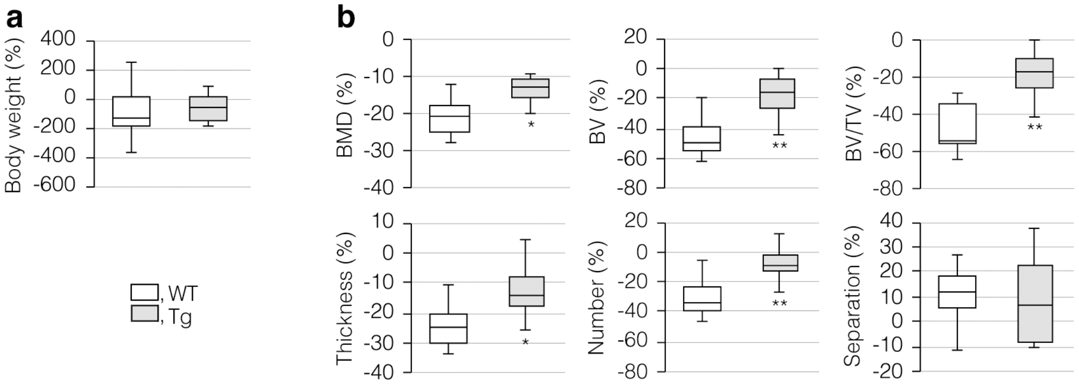

Supplementary Fig. 11. Tg mice protect bone from neurectomy. Ten-week-old male Tg and WT mice were either sham-operated or bilaterally sciatic neurectomized (NX) and analyzed 4 weeks post operation. Data are shown as percent changes of sham-operated mice vs. NX mice (see Fig. 4(**d**)). (**a**) Body weights. (**b**) Bone morphometric parameters of the distal femurs (WT, *n*=7; Tg, *n*=10). **P*<0.05 and ***P*<0.01 by Student's *t*-test.

Supplementary Tables

Supplementary Table 1. Relatively abundant miRNAs in MVs and their possible target genes in correlation with or without osteoclast formation

	ID	Normalized value	Predicted target gene	Effect on osteoclast formation	Reference
	mmu-miR-5126	9.203	Dicer1, Gnl3l, Nedd4l		
	mmu-miR-3963	9.193	Zfp799, Haus2, Dmrta1		
	mmu-miR-5126	9.203	Dicer1, Gnl3l, Nedd4l		
	mmu-miR-466i-5p	8.745	Snap35, Prrc2b, Slc6a6		
	mmu-miR-3960	8.104	NDUFA11, C19orf26, TOR4A	Not reported	
	mmu-miR-574-5p	7.501	Dera, Nusap1, FOXN3	Not reported	
	mmu-miR-1224-5p	7.355	NUP62, WSF2, Smg1	Not reported	
	mmu-miR-3082-5p	7.102	Kif1b, Hs3st3b1, Dusp9		
	mmu-miR-1187	6.731	Ado, Lifr, Igf2		
	mmu-miR-709	6.69	Ctcfl, Myc, Akt1		
	mmu-miR-5100	6.446	EIF5, NPEPPS, SESN3	Not reported	
	mmu-miR-211-3p	6.409	CHAC1, TNFSF9, SET	Not reported	
	mmu-miR-5103	6.356	Gla		
	mmu-miR-699n	6.331	Hdac5, Runx2, TXNIP	Not reported	
	mmu-miR-2861	5.643	RUNX2, HDAC5	Suppression	Kim, J. H., *et al*. *Biochem J*. **436**, 254-262 (2011).
	mmu-let-7b-5p	5.576	CDK6, SLC25A1, RHD10	Suppression	Ogasawara, T., *et al.* *J Bone Miner Res.* **19,** 1128-1136 (2004).
	mmu-miR-494-3p	5.542	PTEN, CDK6, BCL2	Not reported	
	mmu-miR-483-5p	5.492	Socs3, SRF, MAPK3	Activation	Oh, J. H., *et al.* *Cell Signal.* **27,** 2325-2331 (2015).
	mmu-miR-32-3p	5.44	KIF5B, WNK1, VPS4A	Not reported	
✔	mmu-miR-125b-5p	5.432	VDR, CYP24A1, PRDM1	Activation	Nisikawa, K., *et al.* *Proc Natl Acad Sci U S A.* **107,** 3117-3122 (2010).<br>Miyauchi, Y., *et al.* *J Exp Med.* **207,** 751-762 (2010).
✔	mmu-let-7c-5p	5.161	TGFBR1, HMGA2, BCL2	Activation	McGill, C. G., *et al*. *Cell* **109**, 707-718 (2002).
	mmu-miR-1927	5.132	Sin3a, Kcnj5, Sos1		
	mmu-miR-504-3p	5.107	FBX028, BIRC5, SEPT2	Not reported	
	mmu-miR-468-3p	4.964	Hells, Zfp128, Xiap		
	mmu-miR-3473b	4.667	Adamts14, Nav2, Cpox		
	mmu-miR-1895	4.603	Car5b, Lsm11, Rabggta		
	mmu-miR-500-3p	4.581	Mtx3, Impad1, Fam214b		
	mmu-miR-5119	4.475	Gm5148, Zer1, Wdr43		
✔	mmu-miR-199a-3p	4.441	MET, CD44, MTOR	Activation	Suzuki, K., *et al.* *J Bone Miner Res.* **17**, 1486-1497 (2002).
	mmu-let-7i-5p	4.425	IL13, BMP4, IGF1	Not reported	
	mmu-miR-466m-5p	4.409	Zfp157, tns4, Oxsm		
	mmu-miR-699l-5p	4.309			
	mmu-miR-3070b-3p	4.301			
	mmu-miR-672-5p	4.254	Tet1, Epha4, Psd3		
	mmu-miR-721	4.19	Meox2, Tmem25, Snx27		
✔	mmu-miR-21a-5p	4.121	Fasl, Peli1, Pdcd4	Activation	Park, H., *et al*. *J Immunol*. **175,** 7193-7201 (2005).
	mmu-let-7a-5p	4.101	NKIRAS2, NF2, FOXO1	Suppression	Tan, P., *et al.* *Sci Rep*. **5,** 16835 (2015).
	mmu-miR-699e-5p	4.022			

See Table 1 for normalized values and light blue columns. Genes in green have been reported to be directly involved in osteoclast formation (see references). We chose the four miRNAs delineated with check marks.

Supplementary Table 2. The possible effects of predicted targets of miR-125b on osteoclast formation

Predicted target	Validation Method (strong evidence)			Osteoclast formation	Reference (representative)
	Reporter assay	Western blot	qPCR		
BMPR1B	✔				
EIF4EBP1	✔		✔		
HNGA2	✔				
HMGA1	✔				
GLI1	✔				
NKIRAS2	✔	✔	✔		
SMO	✔	✔			
TP53	✔	✔	✔		
VDR	✔	✔	✔		
SGPL1	✔				
BAK1	✔	✔	✔		
ERBB3	✔	✔	✔		
ERBB2	✔	✔	✔		
BMF	✔		✔		
KLF13		✔	✔		
NTRK3	✔	✔			
LIN28A	✔	✔	✔		
CBFB	✔	✔	✔		
AKT1		✔	✔	✔	Sugitani, T. & Hruska, K. A. *J Biol Chem.* **280**, 3583-3589 (2005)
CYP24A1	✔	✔	✔		
RAF1		✔			Li, S. *et al*. *J Clin Invest.* **124,** 5057-5073 (2014)
PRDM1	✔		✔	✔	Miyauchi, Y. *et al*. *J Exp Med.* **207,** 751-762 (2010)<br>Nishikawa, K. *et al*. *Proc Natl Acad Sci USA* **107,** 3117-3122 (2010)
CDKN2A	✔	✔			
KRT7			✔		
GRIN2A	✔		✔		
IRF4	✔		✔	✔	Nakashima, Y. & Haneji, T. *PLoS One* **8**, e72033 (2013)
TP53INP1	✔	✔	✔		
E2F3	✔	✔	✔		
IGF2	✔	✔			
LIN28B	✔	✔	✔		
BAK1	✔	✔	✔		
BBC3	✔	✔	✔		
TEF		✔	✔		
PPP1CA	✔	✔	✔		
PRKRA	✔	✔	✔		
BCL2	✔	✔		✔	Bozec, A. *et al*. *Nature* **454,** 221-225 (2008)
RPS6KA1	✔	✔	✔		Drosatos-Tampakaki, Z. *et al*. *J Bone Miner Res*. **29**, 1183-1195 (2014)
TNFAIP3		✔	✔	✔	de la Rica, L. *et al. Genome Biol.* **5**, 16, 2 (2015)
PIGF	✔	✔	✔		
BCL3	✔				
TBC1D1	✔				
DGAT1	✔				
FGFR2	✔	✔	✔		
ARID3B	✔				
SMAD4	✔	✔		✔	Tasca, A. *et al*. *J Cell Biochem.* **116,** 1350-1360 (2015)
MCL1	✔	✔	✔	✔	Rimondi, E. *et al. Invest New Drugs.* **31**, 780-786 (2013)
IL6R	✔	✔	✔	✔	Gelb, B. D., Edelson, J. G. & Desnick, R. J. *Nature Genet.* **10**, 235-237
STARD13	✔				
ABTB1	✔				
HK2	✔		✔		
E2F2	✔	✔	✔		
MMP13	✔				
MAPK14	✔	✔	✔	✔	Fujita, K. *et al*. *Nat Med.* **18**, 589-594 (2012)
EPO	✔				
MUC1	✔	✔			
NES	✔				
CDH5		✔	✔		
ARID3A	✔	✔	✔		
BCL2L2	✔	✔	✔		
IGF1R	✔	✔	✔		
NCOR2	✔	✔			
PRTG	✔				
EIF5A2	✔	✔	✔		
MXD1	✔	✔	✔		
PIAS3	✔	✔			
PIK3CD	✔	✔	✔		
PCTP	✔	✔	✔		
LIPA	✔	✔	✔		
GSS	✔	✔	✔		

GSS	✔	✔	✔		
IKZF2	✔	✔	✔		
IKZF3	✔	✔	✔		
IKZF4	✔	✔	✔		
ICAM2	✔		✔		
VPS4B	✔				
CCNJ	✔	✔			
ENPEP	✔	✔			
CSNK2A1	✔	✔			
MEGF9	✔	✔			
MAN1B1	✔	✔	✔		
TACSTD2	✔				
EPOR	✔				
AHRR	✔				
SCNN1A	✔	✔	✔		
VPS51	✔				
SIRT7	✔	✔	✔		
DUSP6	✔	✔	✔		
TET2	✔				

Predicted target	Validation Method (less strong evidence)	Osteoclast formation	Reference (representative)
XIAP	Next-generation DNA sequencing	✔	Koka, K. et al. *Endocrinology* **141**, 2995-3005 (2000).
ADRM1	Next-generation DNA sequencing	✔	Kim, T. et al. *Biochem Biophys Res Commun* . **390**, 585-590 (2009).
BACH1	Next-generation DNA sequencing	✔	Hama, M. et al. *Arthritis Rheum* . **64**, 1518-1582 (2012).
CREBBP	Next-generation DNA sequencing	Indirect	Cary, R. L. et al. *J Bone Miner Res* . 28, 1599-1610 (2013).
FUS	Next-generation DNA sequencing	✔	Bronisz, A. et al. *J Biol Chem.* **289**, 326-334 (2014).
PARP1	Next-generation DNA sequencing	Inhibition	Beranger, G. E. et al. *J Bone Miner Res* . **22**, 975-983 (2007).

Predicted targets of miR-125b in light green columns are positively implicated in osteoclast formation. Genes in Ligt yellow columns were used as negative controls for qRT-PCR analysis.

Supplementary Table 3. Primer sets for PCR

Gene	Primers (5' - 3')	GeneBank Accession number *etc.*	Species
Osteocalcin prompter	AACAAGGTGGTTAGACTGCAAAG<br>AACAAGGTGGTTAGACTGCAAAG<br>AAAAGCGGCCGCCATGCTGTTTTGGTTACTAGAGCCT<br>AAAAACTAGTGGTGTCTCGGTGGCTGCGCTGGGCT	BAC clone (RP11-964F7)	Human
βglobin	CTGGTCATCATCCTGCCTTT<br>TTAAGCTTACAAAGAATGGCCACAGG	Transgene in Tg mice	Rabbit
3'UTR of Prdm1	TTACTAGTATGGCTTGGTGAATCAGGG<br>TTAAGCTTACAAAGAATGGCCACAGG	NM_007548.3	Mouse
3'UTR of Prdm1, mutant	CTAGTCACCATAAATGAGGCAAAGACTC<br>TTTTGGTAACATTGGAGTCCTTC		
Adm1	GCCCTCTCATGTGCCAGTT<br>CATTGTTCTGCATGGCTTTG	NM_019822.3	Mouse
Akt1	CCCTTCTACAACCAGGACCA<br>ATACACATCCTGCCACACGA	NM_001165894.1	
Actb	TTTTCCAGCCTTCCTTCTTG<br>ACGGATGTCAACGTCACACT	NM_007393	
Bach1	CAGGGGGACAGTCAGTAGGA<br>AGATGCAGCATGCAATGAAG	NM_007520.2	
Bcl2	TGAGGACCCAATCTGGAAAC<br>CTTGCAATGAATCGGGACTT	NM_009741	
Bcl6	GGCCAGTGAAGCAGAAATGG<br>ACGACCTCACGACCTCGGTA	NM_009744	
Catpk	CTTCCAATACGTGCAGCAGA<br>TCTTCAGGGCTTTCTCGTTC	AK_003425	
Crebbp	TGAACATCATGAACCCAGGA<br>AGCAGCTGTCTCCTCACCAT	NM_001025432.1	
Dcstamp	AGAGAGGCTGACGGGAAACC<br>GGCCCAGAAAGAGGGACTGT	NM_029422	
Fus	CCCTGGCAAGATGGACTC<br>AACTTCAGGAGCCAGGCTAA	NM_139149.2	
Il6r	AGGGTGTGTCGTTCCTGCTA<br>CATCTGAGGCCACTCAGTCA	NM_001310676.1	
Irf4	GGCCCAACAAGCTAGAAAGA<br>CCATGGTGAGCAAACACTTG	NM_013674	
Irf8	GTTCCGTATCCCCTGGAAGC<br>GTCTTCCACGTGGCTGGTTC	BC_005450	
Mafb	TCGTCCCAGGAAGAGCAGAG<br>CGGCTGAGAGCCAGTGTTCT	NM_010658	
Mapk14	CCTTTGAAAGCAGGGACCTT<br>GTGGCACAAAGCTGATGACT	NM_001168513	
Mcl1	TGGGTTTGTGGAGTTCTTCC<br>AAAGCCAGCAGCACATTTCT	NM_008562.3	
Nfatc1	GCCAGTACCAGCGTTTCACG<br>CTGGCTCATTGGTCCACAGG	NM_001164112	
Prdm1	CAGACCTGCAACAAGGGTTT<br>TCGAAGGTGGGTCTTGAGAT	NM_007548	
Rpl32	AGTTCATCAGGCACCAGTCA<br>TGTCAATGCCTCTGGGTTT	NM_172086	
Parp1	AGAGGTTCCACTGGGAACAG<br>GATTCACCTGAGCAATGTCGT	NM_007415.2	
Smad4	ATGGCTATGTGGATCCTTCG<br>CCAAACGTCACCTTCACCTT	NM_008540.2	
Tnfaip3	TGGGAAGGGACACAACTACA<br>GCAGAAACTTCCTCGTCCTC	NM_001166402.1	
Tracp	AACGTCTCTGCACAGATTGC<br>AAGCGCAAACGGTAGTAAGG	BC_012911	
Xiap	CCCAAAGCTCATGGTCAGTT<br>GCTCTCACCTTCCCACTTTG	NM_001301639.1	

Supplementary Table 4. Antibodies for FACS

Cell type	Antinody
Hematopoietic stem cell (HSC)	Cocktail-FITC
	CD117-PE
	CD34-APC
	Sca1-BV421
Macrophage	CD115-Alexa Fluor 488
	CD11b-APC
	F4/80-BV421
Osteoclast precursor	CD115-Alexa Fluor 488
	CD11b-APC
	CD117-PE
B cell	CD19-FITC
	CD45R/B220-PE
T cell	CD3-BV421

Supplementary Methods

Transmission electron microscopy

MC3T3-E1 cells at day 14 on coverslips and their corresponding MV pellets were fixed in 2% glutaraldehyde in 0.1 M cacodylate buffer (pH 7.4) at 4°C overnight. Samples were post-fixed in 1% osmium tetroxide in 0.1 M phosphate buffer (pH 7.4) for 1 h, rinsed, dehydrated, and embedded in epoxy resin. Ultrathin sections were mounted on a copper grid and examined using a JEM-1230S (JEOL, Tokyo, Japan) at 80 kV.

NanoSight analysis

MVs isolated from MC3T3-E1 (5 µL stock solution) were suspended in PBS and subjected to NanoSight analysis (NS300, Quantum Design), according to the manufacturer's instructions.

Histology

Decalcified paraffin sections were stained with hematoxylin and eosin or toluidine blue. Plastic sections were stained with Villanueva.

Sciatic neurectomy (NX)

Bilateral sciatic neurectomy[33] or sham operation was performed on 10-week-old male mice, and mice were fed for additional 4 weeks.

Cocultures

Calvaria cells derived from newborn Tg and WT were seeded at 3,000 cells/cm^2 and maintained with osteogenic medium until osteoid like-nodules were formed as osteoblasts (see the Methods). BMMs were obtained from 10-week-old male Tg and WT mouse femurs and tibiae (see the Methods). Osteoblasts were pretreated 1,25-dihydroxyvitamin D_3 (10 nM) and prostaglandin E_2 (1 µM) for a day and subsequently overlaid with or without matched- and unmatched-mouse line BMMs (12,000 cells/cm^2) and maintained until multinucleated cells are formed[1]. After fixation with 4% PFA in PBS, TRAP staining was performed.

MV labeling and uptake

MC3T3-E1 cells were incubated with Vybrant® DiI cell-labeling D-282 solution (5 µM final concentration; Life Technologies) at 37°C for 30 min according to the manufacturer's instructions. MVs were isolated as described in the Methods. MC3T3-E1 cells and RAW-D cells were grown at 3,000 cells/cm^2 and 2,200 cells/cm^2, respectively, in glass bottom dishes (Greiner Bio-One,) for 1 day, and then treated with labeled MVs (2 µg/mL protein). Time-lapse imaging was conducted using the Incucyte™ Zoom system (Essen BioScience).

Alkaline phosphatase (ALP) activity

ALP activity in aliquots (protein levels) of lysates were measured using a LabAssay™ ALP kit (WAKO). Aliquots of samples (2 µg protein) were subjected to assays.

Western blotting

Aliquots of lysates (MVs, 2µg protein; cells, 10 µg protein) were resolved on SDS-PAGE under reducing conditions and electroblotted onto nitrocellulose membranes (Hybond-ECL™; Millipore). Membranes were incubated with 0.5% casein in Tris-buffered saline including

0.025% Tween 20 and probed with primary antibodies against ALPL, ANX5 and TNFRSF11B (×500 each, R&D Systems), PRDM1 (×1,000, Cell Signaling Technology), IRF8 and MAFB (×500 each, Proteintech) at 4°C overnight. Membranes were then treated with horseradish peroxidase-conjugated secondary antibody (Santa Cruz Biotechnology), followed by chemiluminescence detection (Lumi-Light^{PLUS}, Roche Diagnostics).

FACS analysis
Bone marrow cells were assessed by FACS, as described in the Methods. See a panel of antibodies used (BioLegend) (Supplementary Table 4).

Statistical analysis
Statistical differences were evaluated as described in the Methods.

Supplementary Reference

1. Marino, S., Logan, J. G., Mellis, D. & Capulli, M. Generation and culture of osteoclasts. *BoneKEy Reports* **3**, 570 (2014).
