# Supplementary material for: The matrix vesicle cargo miR-125b accumulates in the bone matrix, inhibiting bone resorption in mice
Source: Commun Biol. 2020 Jan 16;3:30. doi: 10.1038/s42003-020-0754-2 (PMC6965124; doi:10.1038/s42003-020-0754-2)
Supplement: Supplementary file 2 — Description of Additional Supplementary Files [file 42003_2020_754_MOESM2_ESM.docx]

**Description of Additional Supplementary Files**

Supplementary Data 1: Source data.
All source data underlying the main figures are available in Supplementary Data 1.

Supplementary Data 2: miRNA array data.
176 miRNAs in MVs isolated from the ECM of MC3T3-E1 cell cultures.
