# Supplementary material for: The matrix vesicle cargo miR-125b accumulates in the bone matrix, inhibiting bone resorption in mice
Source: Commun Biol. 2020 Jan 16;3:30. doi: 10.1038/s42003-020-0754-2 (PMC6965124; doi:10.1038/s42003-020-0754-2)
Supplement: Supplementary file 5 — Reporting Summary [file 42003_2020_754_MOESM5_ESM.pdf]

natureresearch

Corresponding author(s): Yuji Yoshiko

Last updated by author(s): 2019/12/9

Reporting Summary

Nature Research wishes to improve the reproducibility of the work that we publish. This form provides structure for consistency and transparency in reporting. For further information on Nature Research policies, see Authors & Referees and the Editorial Policy Checklist.

Statistics

For all statistical analyses, confirm that the following items are present in the figure legend, table legend, main text, or Methods section.

n/a	Confirmed	
☐	☒	The exact sample size (*n*) for each experimental group/condition, given as a discrete number and unit of measurement
☐	☒	A statement on whether measurements were taken from distinct samples or whether the same sample was measured repeatedly
☐	☒	The statistical test(s) used AND whether they are one- or two-sided<br>*Only common tests should be described solely by name; describe more complex techniques in the Methods section.*
☐	☒	A description of all covariates tested
☒	☐	A description of any assumptions or corrections, such as tests of normality and adjustment for multiple comparisons
☐	☒	A full description of the statistical parameters including central tendency (e.g. means) or other basic estimates (e.g. regression coefficient) AND variation (e.g. standard deviation) or associated estimates of uncertainty (e.g. confidence intervals)
☐	☒	For null hypothesis testing, the test statistic (e.g. *F*, *t*, *r*) with confidence intervals, effect sizes, degrees of freedom and *P* value noted<br>*Give P values as exact values whenever suitable.*
☒	☐	For Bayesian analysis, information on the choice of priors and Markov chain Monte Carlo settings
☒	☐	For hierarchical and complex designs, identification of the appropriate level for tests and full reporting of outcomes
☒	☐	Estimates of effect sizes (e.g. Cohen's *d*, Pearson's *r*), indicating how they were calculated

Our web collection on statistics for biologists contains articles on many of the points above.

Software and code

Policy information about availability of computer code

Data collection	Microsoft Excel, ImageJ (NIH), NRecon (Bruker), CTVox (Bruker), Image Lab (Bio-Rad), StepOne Plus Real-Time PCR system (ThermoFisher Scientific), CellSens (Olympus Life Science), GloMax Explorer System (Promega), BD FACSDiva (BD), LAS X (Leica Microsystems), NanoSight (Quantum Design)
Data analysis	Microsoft Excel, ImageJ (NIH), CTAn (Bruker), GeneSpring GX (Agilent Technologies), JMP (SAS)

For manuscripts utilizing custom algorithms or software that are central to the research but not yet described in published literature, software must be made available to editors/reviewers. We strongly encourage code deposition in a community repository (e.g. GitHub). See the Nature Research guidelines for submitting code & software for further information.

Data

Policy information about availability of data

All manuscripts must include a data availability statement. This statement should provide the following information, where applicable:

- Accession codes, unique identifiers, or web links for publicly available datasets
- A list of figures that have associated raw data
- A description of any restrictions on data availability

Our data in this study are available within the paper.

Field-specific reporting

Please select the one below that is the best fit for your research. If you are not sure, read the appropriate sections before making your selection.

☒ Life sciences ☐ Behavioural & social sciences ☐ Ecological, evolutionary & environmental sciences

For a reference copy of the document with all sections, see nature.com/documents/nr-reporting-summary-flat.pdf

Life sciences study design

All studies must disclose on these points even when the disclosure is negative.

Sample size	For in vitro studies; n=3 for gene expression and protein levels, microarray, fluorescence labeling of cells, luciferase activities, ALP/von Kossa staining in cell cultures, n=6 for counting cells and resorption pit formation.<br>As for in vivo studies; n=5~7 for histological and bone morphometric analyses, n=6 for body weight changes, n=8 for FACS analysis, n=8 for ovariectomy and n=7~10 for neurectomy. A representative of three independent experiments is shown in other figures such as electron microscopy and NanoSight analysis.
Data exclusions	No data were excluded from the analyses, except for Western blotting.
Replication	All attempts at replication were successful.
Randomization	In ovariectomy and neurectomy experiments, mice were randomized based on body weight. Groups in culture plates were equally arranged.
Blinding	The samples were encrypted and associated with the code number.

Reporting for specific materials, systems and methods

We require information from authors about some types of materials, experimental systems and methods used in many studies. Here, indicate whether each material, system or method listed is relevant to your study. If you are not sure if a list item applies to your research, read the appropriate section before selecting a response.

Materials & experimental systems

n/a	Involved in the study
☐	☒ Antibodies
☐	☒ Eukaryotic cell lines
☒	☐ Palaeontology
☐	☒ Animals and other organisms
☒	☐ Human research participants
☒	☐ Clinical data

Methods

n/a	Involved in the study
☒	☐ ChIP-seq
☐	☒ Flow cytometry
☒	☐ MRI-based neuroimaging

Antibodies

Antibodies used	Goat anti-rabbit IgG Alexa Fluor 594 (Life Technology, Cat #A11303, Lot #1099502), Donkey anti-goat IgG-HRP (Santa Cruz Biotechnology, Cat #sc-2056, Lot #C1207), Donkey anti-rabbit IgG-HRP (Santa Cruz Biotechnology, Cat #sc2077, Lot #K0810), Blimp-1/PRDI-BF1(C14A4) rabbit monoclonal (Cell Signaling Technology, Clone #C14A4, Cat #91155, Lot #6), Actin (I-19) goat polyclonal (Santa Cruz Biotechnology, Cat #sc-1616, Lot #F2106), MAFB rabbit polyclonal (Proteintech, Cat #20189-1-AP, Lot #00013765), IRF8 rabbit polyclonal (Proteintech, Cat #18977-1-AP, Lot #00010445), Annexin V rabbit polyclonal (Proteintech, Cat #11060-1-AP, Lot #00001360), ALPL rabbit polyclonal (Proteintech, Cat #11187-1-AP, Lot #00001622), TNFRSF11B goat polyclonal (R&D, Cat #AF459, Lot #DRM0518041). AGO2 antibodies were included in a MagCapture micoRNA Isolation kit, Mouse Ago2 (WAKO, Cat #297-74201, Lot #CTQ4309), Please see the Flow Cytometry section for antibodies used for FACS (validated for FACS).
Validation	Blimp-1/PRDI-BF1(C14A4) rabbit monoclonal (Specificity, human and mouse; Tested application, WB, IF, IP), Actin (I-19) goat polyclonal (Specificity, human, mouse, rat; Tested application, WB), MAFB rabbit polyclonal (Specificity, human, mouse, rat; Tested application, WB), IRF8 rabbit polyclonal (Specificity, human, mouse, rat; Tested application, WB, IHC), Annexin V rabbit polyclonal (Specificity, human, mouse, pig; Tested application, WB, IP, IHC, FACS), ALPL rabbit polyclonal (Specificity, human, not confirmed for mouse; Tested application, WB, IHC, FACS), TNFRSF11B goat polyclonal (Specificity, mouse; Tested application, WB, ELISA, IHC).

Eukaryotic cell lines

Policy information about cell lines

Cell line source(s)	MC3T3-E1 cells, RIKEN BRC (Japan); RAW-D cells, Kyushu University (Dr. Kukita, Japan)
Authentication	Authenticated by RIKEN and Kyushu University.
Mycoplasma contamination	Cell lines used were tested to be negative for mycoplasma contaminations.
Commonly misidentified lines (See ICLAC register)	N/A

Animals and other organisms

Policy information about studies involving animals; ARRIVE guidelines recommended for reporting animal research

Laboratory animals	C57BL/6J mice, from newborn to 15-week-old males and /or females; ddY mice, 8-week-old males; Wistar rats, time-pregnant females.
Wild animals	N/A
Field-collected samples	N/A
Ethics oversight	N/A

Note that full information on the approval of the study protocol must also be provided in the manuscript.

Flow Cytometry

Plots

Confirm that:

☐ The axis labels state the marker and fluorochrome used (e.g. CD4-FITC).

☐ The axis scales are clearly visible. Include numbers along axes only for bottom left plot of group (a 'group' is an analysis of identical markers).

☒ All plots are contour plots with outliers or pseudocolor plots.

☒ A numerical value for number of cells or percentage (with statistics) is provided.

Methodology

Sample preparation	Bone marrow cells were prepared from femoral and tibial marrow cells of 10-week-old mice. Cells were passed through a cell strainer (70 µm, Falcon®), centrifuged, and the resultant cell pellets were resuspended in ACK lysis buffer (0.15 M NH4Cl, 0.01 M KHCO3, and 1 mM Na2EDTA; pH7.4) (hemolysis). After gentle agitation for 2 min, cell suspensions were centrifuged and rinsed. Resuspended cells (4x10^5 cells in 100 µL of 0.5% BSA in PBS) were treated with TruStain fcX for 5 min on ice, followed by incubation with labeled antibodies or isotype controls for 60 min ad 4°C. Cells were then treated with 1 mM EDTA and 2% BSA in PBS, centrifuged and resuspended with 0.5% BSA in PBS. Antibodies used in this study were as follows; FITC anti-mouse Lineage Cocktail (BioLegend, Cat #420403, Lot #B159364), Brilliant Violet 421 anti-mouse Ly-6A/E (Sca-1) (BioLegend, Cat #133301, Lot #B219976), Anti-mouse CD117 (c-kit) PE (eBioscience, Cat #12-1171, Lot #E01443-1633), APC mouse anti-human CD34 (BD Biosciences, Cat #560940, Lot #5113938), Alexa Fluor 488 anti-mouse CD115 (CSF-1R) (BioLegend, Cat #155512, Lot #B225234), APC rat anti-mouse/human CD11b (BD Biosciences, Cat #553312, Lot #7039760), Brilliant Violet 421 anti-mouse F4/80 (BioLegend, Cat #123137, Lot #B226240), FITC anti-mouse CD19 (BioLegend, Cat #115505, Lot #B215594), PE anti-mouse/human CD45R/B220 (BioLegend, Cat #103207, Lot #B199460), Brilliant Violet 421 anti-mouse CD3 (BioLegend, Cat #100227, Lot #B225202), and TruStain fcX (anti^mouse CD16/32) (BioLegend, Cat #101320, Lot #B247950).
Instrument	LSRFortessa X-20 (BD Biosciences)
Software	BD FACSDiva (BD Biosciences)
Cell population abundance	According to the References
Gating strategy	According to the References

☒ Tick this box to confirm that a figure exemplifying the gating strategy is provided in the Supplementary Information.
